# High titer and yield ethanol production from undetoxified whole slurry of Douglas-fir forest residue using pH profiling in SPORL

**DOI:** 10.1186/s13068-015-0205-3

**Published:** 2015-02-15

**Authors:** Jinlan Cheng, Shao-Yuan Leu, JY Zhu, Rolland Gleisner

**Affiliations:** Jiangsu Provincial Key Lab of Pulp and Paper Science and Technology, Nanjing Forestry University, Nanjing, China; USDA Forest Service, Forest Products Laboratory, Madison, WI 53719 USA; Department of Civil and Environmental Engineering, Hong Kong Polytechnic University, Kowloon, Hong Kong

**Keywords:** Forest residue, Enzymatic hydrolysis and fermentation, High solids processing, Fermentation inhibitors, High-titer biofuel

## Abstract

**Background:**

Forest residue is one of the most cost-effective feedstock for biofuel production. It has relatively high bulk density and can be harvested year round, advantageous for reducing transportation cost and eliminating onsite storage. However, forest residues, especially those from softwood species, are highly recalcitrant to biochemical conversion. A severe pretreatment for removing this recalcitrance can result in increased sugar degradation to inhibitors and hence cause difficulties in fermentation at high solid loadings. Here, we presented high titer ethanol production from Douglas-fir forest residue without detoxification. The strong recalcitrance of the Douglas-fir residue was removed by sulfite pretreatment to overcome the recalcitrance of lignocelluloses (SPORL). Sugar degradation to inhibitors was substantially reduced using a novel approach of “pH profiling” by delaying acid application in pretreatment, which facilitated the simultaneous enzymatic saccharification and fermentation of undetoxified whole slurry at a solid loading of 21%.

**Results:**

“pH profiling” reduced furan production by approximately 70% in using SPORL pretreating Douglas-fir forest residue (FS-10) comparing with the control run while without sacrificing enzymatic saccharification of the resultant substrate. pH profiling also reduced carbohydrate degradation. The improved carbohydrate yield in pretreated solids and reduced fermentation inhibitors with pH profiling resulted in a terminal ethanol titer of 48.9 ± 1.4 g/L and yield of 297 ± 9 L/tonne FS-10, which are substantially higher, i.e., by 27% in titer and by 38% in yield, than those of a control SPORL run without pH profiling.

**Conclusions:**

Economical and large-volume production of commodity biofuels requires the utilization of feedstocks with low value (therefore low cost) and sustainably producible in large quantities, such as forest residues. However, most existing pretreatment technologies cannot remove the strong recalcitrance of forest residues to produce practically fermentable high titer sugars. Here, we demonstrated a commercially scalable and efficient technology capable of removing the strong recalcitrant nature of forest residues using “pH profiling” together with “low temperature SPORL”. The resultant pretreated whole slurry of a Douglas-fir forest residue using this technology can be easily processed at high solids of 21% without detoxification to achieve a high ethanol yield of 297 L/tonne at 48.9 g/L.

**Graphical Abstract:**
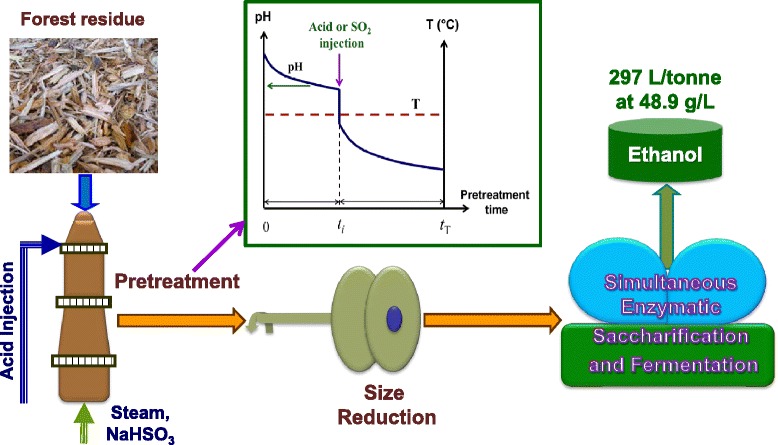
Graphic table of content.

**Electronic supplementary material:**

The online version of this article (doi:10.1186/s13068-015-0205-3) contains supplementary material, which is available to authorized users.

## Background

Lignocellulosic biomass, as a structural material, has natural resistance to enzymatic deconstruction for production of fermentable sugars. Pretreatment, a step to remove this recalcitrance, increases the cellulose accessibility to cellulase for efficient saccharification of polysaccharides in lignocelluloses. Most promising and commonly practiced acidic pretreatments, such as dilute acid [1,2], sulfite pretreatment to overcome recalcitrance of lignocellulose (SPORL) [2,3], organosolv [4], and SO_2_-catalyzed steam explosion [5,6], however, can degrade sugars to undesirable compounds such as furans. These pretreatments also convert acetyl groups on the hemicellulose backbone into acetic acid. The undesirable sugar degradation products and acetic acid are inhibitive to many microbes and catalysts, such as *Saccharomyces cerevisiae*, during conversion of sugars to biofuel and bioproducts. Removal of the inhibitive compounds through detoxification steps is possible, but at additional costs, which negatively affects production economics.

Reduced sugar degradation during pretreatment is desirable for high solid processing without detoxification to achieve high biofuel titer. Low-temperature pretreatment can reduce inhibitor formation. An early study arbitrary reduced the reaction duration to result in a lower pretreatment severity to reduce degradation [7], but at the expense of lower sugar yield. Additional processing steps such as alkali extraction, disk refining, and xylanase supplementation were necessary to maintain enzymatic saccharification efficiency. Recently, we conducted low-temperature pretreatment while maintaining reaction severity measured by a combined hydrolysis factor (CHF) based on hemicellulose dissolution [8]. We used a constant CHF to determine the extended reaction time at a reduced pretreatment temperature. We were able to reduce furan formation by approximately 50% in pretreating Douglas-fir wood chips while maintaining similar high (>90%) cellulose enzymatic saccharification efficiency by reducing the pretreatment temperature from 180°C to 165°C [8]. Further reduction in furan formation is possible by using an even lower temperature but requires a much longer pretreatment time, which reduces production capacity [9].

Forest residue is one of the most cost-effective feedstock for biofuel production based on a recent study by the US National Academy of Sciences [10]. It can be sustainably produced in large quantities in North America and various regions of the globe [11-13]. Furthermore, it has relatively high bulk density and can be harvested year round to eliminate onsite storage, both of which are advantages over agriculture residue and herbaceous biomass for improving the supply chain logistics and reducing transportation costs [14,15]. However, forest residues have high lignin content due to the presence of bark and juvenile wood which can increase recalcitrance to biochemical conversion. A severe pretreatment required to remove this recalcitrance can produce increased sugar degradation to furan even using the low-temperature pretreatment strategy [16]. Because low furan formation can not only reduce sugar loss but also facilitate fermentation to increase fermentation yield especially at high solid loadings [17], improving pretreatment with low furan formation is always desirable and needed.

**Figure 1:**
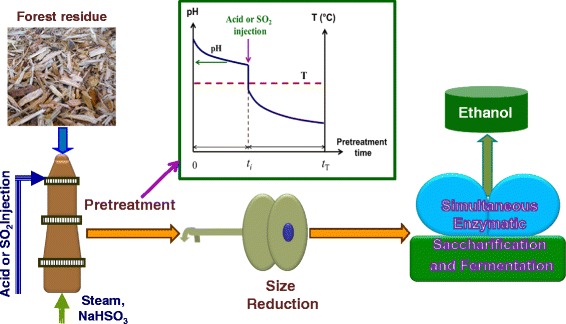
**A schematic experimental block flow diagram with the illustration of the “pH profiling” concept in SPORL for high titer and yield ethanol production without detoxification from a Douglas-fir forest residue.**

Here, we demonstrated the “pH profiling” concept in SPORL [3] to substantially reduce furan formation to achieve high titer and high ethanol yield from SPORL pretreated whole slurry of a Douglas-fir forest residue without solid and liquid separation, solid washing, and detoxification. In this concept, the application of acid such as SO_2_ or H_2_SO_4_ was delayed (the amount of acid applied was unchanged) to purposely control the time-dependent pH profile during pretreatment as shown by the insert plot in Figure 1. The concept is based on two fundamental understandings: (1) the role of acid and sulfite on hemicellulose dissolution [2], sugar degradation, delignification, and sulfonation [18], and deacetylation [19] and (2) the effects of hemicellulose dissolution and delignification on improving cellulose accessibility and cellulose enzymatic saccharification [20-24]; specifically, the quantitative contributions of delignification and hemicellulose removal to enzymatic digestibility of softwood species and forest residues [8,20]. The improved delignification due to increased pH in the early stage of pretreatment compensated for the reduced hemicellulose dissolution as a result of delayed acid application in a “pH profiling” pretreatment. Therefore, equivalent enzymatic saccharification can be achieved but at much low sugar degradation and furan formation. SPORL was chosen for demonstrating the “pH profiling” concept because delignification can be achieved at acidic conditions using sulfite which can reduce the pH range and therefore acid application in addition to the robust performance of SPORL for bioconversion of softwoods and forest residues [16,25]. The concept is similar to two-stage sulfite pulping [26-28] but with the purpose to reduce sugar degradation rather to improve pulp yield. When implementing “pH profiling” for pretreatment, lower chemical loadings, higher temperatures, and shorter residence time than those in sulfite pulping are used as demonstrated here. Multiple or continuous acid application can also be implemented.

## Results and discussions

### Upgrade of Douglas-fir forest residue by physical fractionation

The results from the present study indicated that fractionation was effective to reduce bark content of the Douglas-fir residue harvested using grinding. Visual observation indicted that the small fractions had darker colors and contained more bark than the large fractions (Additional file 1: Figure S1). The smallest fraction (<3.2 mm) was only approximately 5% of the total mass (Additional file 1: Figure S2a) but contained 20% of the total bark in the harvested residue (Additional file 1: Figure S2b). Therefore, rejecting the smallest fraction (<3.2 mm) through screening can selectively reduce the bark and therefore lignin content to result in an upgraded residue. The resultant Douglas-fir forest residue labeled as FS-10 had a bark content of 4.7% compared with 5.9% in the as-harvested residue to result in a lower lignin and higher carbohydrate contents of 29.3% and 56.4%, respectively, than the 30.5% and 50.3% of the as-harvested residue (Table 1). Upgrading through screening increased the carbohydrate content by 12%.

**Table 1:** **Chemical compositions of the as harvested and screening upgraded (FS-10) Douglas-fir forest residue**

**Untreated forest residue**	**Bark (%)**	**Glucan (%)**	**Xylan (%)**	**Mannan (%)**	**K Lignin (%)**	**G + X + M** ^**a**^ **(%)**
As harvested	5.9	38.4	4.4	7.5	30.5	50.3
Screening upgraded (FS-10)	4.7	41.0	5.7	9.7	29.3	56.4

^a^Major carbohydrate = glucan (G) + xylan (X) + mannan (M).

### FS-10 cell wall modification by pretreatments with and without pH profiling

Four SPORL pretreatments of Douglas-fir forest residue FS-10 were conducted with one control, i.e., no active pH profiling, and three pH profiling runs. The key wood component yields from the pretreated washed solids and pretreatment hydrolysates (spent liquors) are listed in Table 2. The sample labels txxA4B12 represents acid application delay time *t*_i_ = xx, sulfuric acid loading of 4 mL/L and sodium bisulfite charge on wood 12 wt.%, respectively. Therefore, t0A4B12 is the control run and the rests are pH profiling runs. As expected, the pH profiling runs increased delignification due to delayed acid application, i.e., lignin yield in washed solids was reduced from 171 g/kg for the control run to approximately 130 g/kg for the pH profiling runs. pH profiling reduced hemicellulose removal. Xylan and mannan yields were increased from 14 and 16 to approximately 21 and 24 g/kg on average of three pH profiling runs, respectively. pH profiling also slightly increased glucan yield from the washed solids which resulted in a lower glucose yield in the spent liquor. The increased carbohydrate yields in the solid substrates potentially can result in increased monomeric sugar recovery through enzymatic saccharification, beneficial to increasing overall sugar yield. pH profiling also reduced monomeric xylose and mannose yields in the spent liquor. However, xylose yield as percentage of dissolved xylan was remained approximately 40% while mannose yield as percentage of dissolved mannan was reduced from approximately 67% to 45% perhaps due to the incomplete hydrolysis as a result of delayed acid application. A substantial amount of dissolved xylan and mannan are expected in the form of oligo-xylose and oligo-mannose, respectively, in the pretreatment spent liquor. The reduction in furan formation by pH profiling was apparent (Table 2) as further discussed in the next section. However, the delay time in acid injection among the three pH profiling runs had minimal effects on component mass yields.

**Table 2:** **Comparisons of yields (per kg of FS-10) of key wood components in the recovered solids and liquid hydrolysate from SPORL pretreatments at 165°C for 75 min with and without pH profiling**

	**Washed solids (g)**					**Pretreatment hydrolysate (spent liquor) (g) (only monomeric sugars were reported)**							
**Pretreated sample** ^**a**^	**Glucan**	**Xylan**	**Mannan**	**K lignin**	**Solids yields** ^**b**^	**Glucose as glucan**	**Xylose as xylan**	**Mannose as mannan**	**K lignin** ^**c**^	**Furfural as pentosan**	**HMF as hexosan**	**Yield** ^**d**^	**Total yield**
t0A4B12	388.4	13.7	15.8	170.6	612.7	21.8	17.7	54.6	120.3	5.0	9.3	228.7	841.4
t25A4B12	405.6	22.7	23.7	130.1	603.9	10.0	13.5	32.8	160.8	2.2	4.0	222.9	826.8
t35A4B12	410.7	20.4	21.8	137.7	616.5	15.7	15.1	38.2	153.2	2.0	3.8	228.0	844.5
t45A4B12	406.5	21.0	25.7	131.9	622.3	9.1	14.2	30.9	159.0	2.0	2.9	218.1	840.4

^a^txx stands for acid injection time in min, A4 stands for 4 mL of sulfuric acid in 1 L of initial pretreatment solution, and B12 stands for 12 wt.% of sodium bisulfite charge on wood.

^b^As measured after disk milling.

^c^Based on balance of lignin.

^d^Sum of listed pretreatment hydrolysate components.

### Effects of pH profiling on furan and acetic acid formation

**Figure 2:**
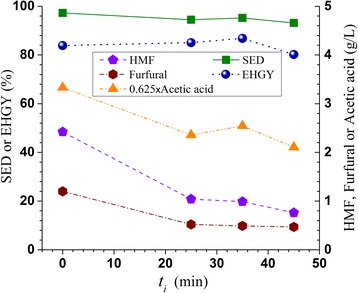
**Effects of acid injection delay time** ***t***_**i**_ **in pretreating a Douglas-fir forest residue on resultant substrate enzymatic digestibility (SED), enzymatic hydrolysis glucose yield (EHGY), HMF, furfural, and acetic acid formation.**

To illustrate the effectiveness of pH profiling in reducing the formation of fermentation inhibitors such as furan and acetic acid, the hydroxymethyl furfural (HMF), furfural, and acetic acid concentrations in the pretreatment spent liquor was plotted against the acid injection delay time *t*_i_ as shown in Figure 2. HMF, furfural, and acetic acid concentrations in the pretreatment spent liquor were reduced from approximately 2.5, 1.2, and 5.3 g/L, respectively, for the control run to approximately 0.8, 0.5, and 3.5 g/L, or by approximately 70%, 60%, and 35% when using the pH profiling technique. The reductions of furan formation resulted from improved carbohydrate preservation (Table 2) due to delayed acid application. The delayed acid application might also reduce the degree of deacetylation reaction to result in low acetic acid production. The results also suggested that acid injection time *t*_i_ did not substantially affect the reductions in furan and acetic acid formation for the studied *t*_i_ range between 25 to 45 min.

### Effect of pH profiling on enzymatic saccharification of pretreated FS-10 solids

**Figure 3:**
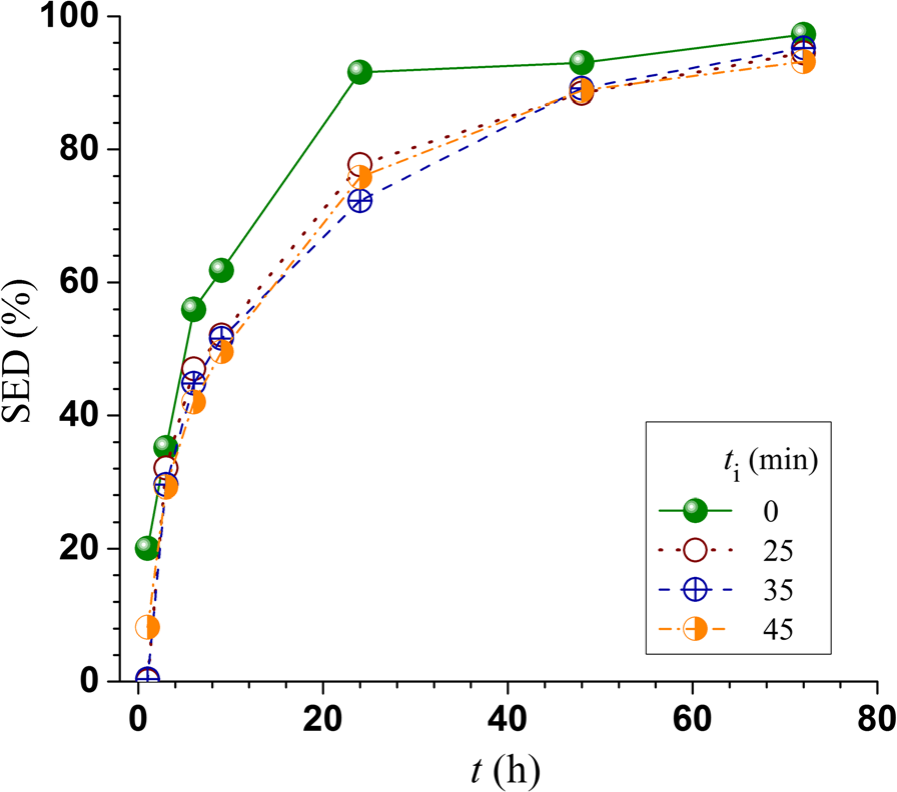
**Effect of acid injection delay time** ***t***_**i**_ **in pretreating a Douglas-fir forest residue on time-dependent resultant substrate enzymatic digestibility (SED).**

The effects of pH profiling on pretreated FS-10 substrate enzymatic digestibility (SED), defined as the percentage of substrate glucan enzymatically saccharified to glucose, and enzymatic hydrolysis glucose yield (EHGY), defined as the percentage of wood glucan recovered as glucose through enzymatic hydrolysis alone, can be observed from Figure 2. It appears that SED was not negatively affected by pH profiling considering that the measurement errors in enzymatic hydrolysis were approximately 2% (not shown for clarity). The results suggested that improved delignification in the pH profiling runs compensated for the reduced hemicellulose removal (Table 2) to achieve similar SED. Similarly, EHGY was not substantially affected by pH profiling. The increased glucan recovery from the solid substrates as discussed previously (Table 2) compensated for the slight reduction in SED by pH profiling to maintain the same level of EHGY. The time-dependent SED data showed that pH profiling reduced the rate of saccharification, but the terminal saccharification efficiency after 72 h was approximately the same as that of the control run (Figure 3).

### Comparisons of high solid fermentation among control and pH profile runs

**Figure 4:**
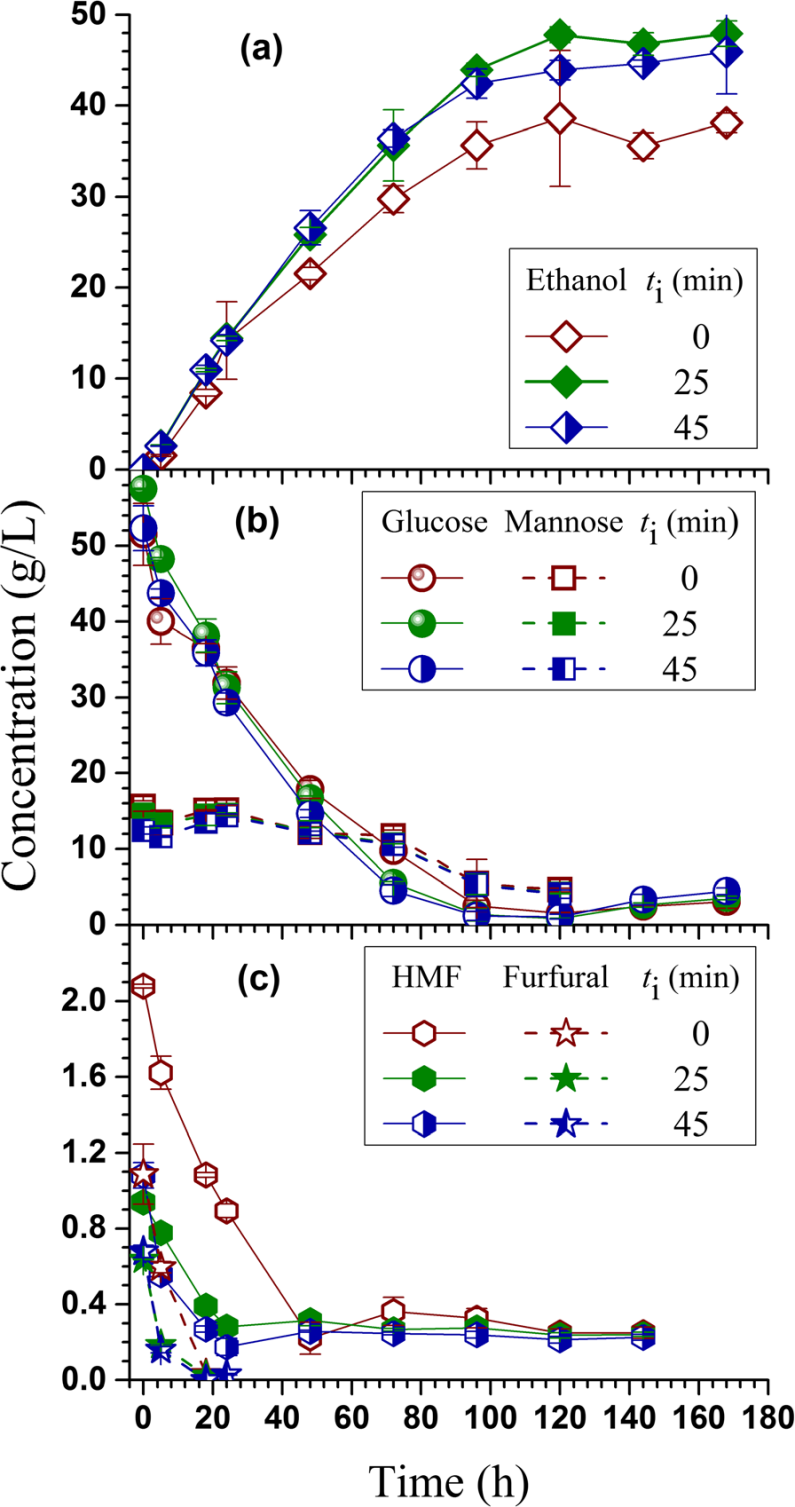
**Comparisons of time-dependent (a) ethanol production, (b) sugar consumptions, (c) and furan metabolization during high solid fermentation of the SPORL pretreated Douglas-fir forest residue with and without pH profiling.**

Quasi-simultaneous enzymatic saccharification and fermentation (Q-SSF) of the pretreated whole slurries from the control and two pH profiling runs were conducted at 21 wt.% total solid loading (approximately water insoluble solid loading of 15 wt.%). The low-temperature pretreatment of 165°C allowed Q-SSF without detoxification even for the control run, perhaps due to sufficient yeast loading at initial optical density (600 nm) of 5 (Figure 4a) [17,29]. The reduced furan formation in the two pH profile runs facilitated fermentation as can be seen from the increased glucose consumption and ethanol productivity in the first 72 h (Table 3), which resulted in higher final terminal ethanol concentrations and yields (Table 3, Figure 4a). The improved carbohydrate preservation on solids (Table 2) also contributed to the higher terminal ethanol titer and yield for the two pH profiling runs. This can be seen from the higher initial glucose concentration for the pH profiling run with acid injection delay time *t*_i_ = 25 min (Figure 4b), though the increase in initial glucose concentration for the pH profiling run with acid injection delay time *t*_i_ = 45 min was insignificant (Figure 4b) due to slightly reduced saccharification efficiency (Figure 3). The continuous enzymatic saccharification throughout the entire Q-SSF process as can be seen from the increase in glucose concentration even after 144 h of fermentation suggests the importance of improving carbohydrate preservation in solids to ethanol production. A terminal ethanol titer of 48.9 g/L and yield 0.494 g/g sugar FS-10 was achieved for the pH profiling run with acid application delay time *t*_i_ = 25 min compared with 38.6 g/L and yield of 0.390 g/g sugar for the control run (Table 3), or an increase over 25% in titer and 27% in yield. It was noticed in Figure 4a that the control run had a very high standard deviation at the peak ethanol concentration or 120 h. Examining the time-dependent ethanol concentration data for the two duplicate fermentation runs (not shown) indicated that run II had a large spike at 120 h followed by a sudden decrease to the same ethanol level in run I. However, both runs showed very similar time-dependent glucose consumption behavior with no abrupt changes. Furthermore, glucose was nearly consumed after 120 h, suggesting no support for the ethanol spike observed in run II. We believe that the large standard deviation in ethanol at 120 h was due to sampling in a nonuniform slurry. Using the HMF concentration in the control run as the inhibitor tolerance limit, an estimated solid loading of approximately 38% can be implemented using pH profiling pretreated whole slurry of FS-10 without detoxification. Considering the effect of other inhibitors on fermentation, a realistic solid loading of approximately 30% may be implemented. This is a substantial advantage in terms of achieving high ethanol titer and reducing water usage in processing. With reduced inhibitor concentration, one can also reduce yeast loading to achieve good ethanol productivity as we demonstrated previously [17].

**Table 3:** **Comparisons of fermentation performance among pretreatments with and without pH profiling.**

	**Control (** ***t***_**i**_ **= 0)**	**pH profiling (** ***t***_**i**_ **= 25 min)**	**pH profiling (** ***t***_**i**_ **= 45 min)**
Average fermentation performance measure (g/L/h)			
Glucose consumption (72 h)	−0.54 ± 0.06	−0.70 ± 0.07	−0.64 ± 0.05
Ethanol productivity (72 h)	0.42 ± 0.03	0.50 ± 0.02	0.51 ± 0.02
HMF metabolization (24 h)	−0.05 ± 0.002	−0.03 ± 0.002	−0.02 ± 0.006
Maximal ethanol production			
Terminal ethanol concentration (g/L)	38.6 ± 7.5	48.9 ± 1.4	45.9 ± 4.6
Ethanol yield (g g/sugar)^a^	0.390 ± 0.076	0.494 ± 0.014	0.460 ± 0.046
Ethanol yield (L/tonne wood)^b^	215 ± 42	297 ± 9	259 ± 26
Ethanol yield (% theoretical)^c^	52.8 ± 10.3	73.1 ± 2.1	63.7 ± 6.4

^a^Based on the total of glucan, mannan, and xylan in the pretreated-solids and glucose, mannose, and xylose in the pretreatment spent liquor.

^b^Calculated from measured amount of ethanol yield from the amount of pretreated whole slurry used in fermentation and the yield of whole slurry from pretreatment.

^c^Theoretical yield (406 L tonne wood^−1^) based on total glucan, mannan, and xylan in the untreated forest residue of FS10.

The acid injection time did not substantially affect glucose consumption in fermentation (Figure 4b, Table 3) because both runs produced similar level of inhibitors (Figure 2, Table 2). Similarly, acid injection time did not substantially affect mannose consumption either (Figure 4b). Mannose consumption was slow in the first 24 h most likely due to the presence of inhibitors. Mannose was not completely consumed after 120 h fermentation. Xylose fermentation was negligible with xylose consumption of only approximately 10% (not shown) though the strain YRH400 is capable of fermenting xylose, in agreement with a previous study [25] using the same strain fermenting a whole slurry of lodgepole pine with a low xylose concentration. The strain YRH400 was able to metabolize both HMF and furfural (Figure 4c), in agreement with a previous study [25]. However, a longer metabolization time was needed for the control run than those for the two pH profiling runs (Figure 4c).

When comparing the two fermentation runs using pH profiling pretreated whole slurry of FS-10, a longer acid application delay time of *t*_i_ = 45 min slightly reduced terminal ethanol concentration (45.9 g/L) as well as ethanol yield (0.460 g/g sugar) compared with those achieved from *t*_i_ = 25 min run of 48.9 g/L and (0.494 g/g sugar), respectively, (Table 3). This is in agreement with the slightly reduced SED and EHGY (Figure 2). The ethanol yield and titer from pH profiling run with *t*_i_ = 45 min run were still respectively higher than those from the control run, i.e., 38.6 g/L and 0.390 g/g sugar.

### Overall mass balance in ethanol production with pH profiling

**Figure 5:**
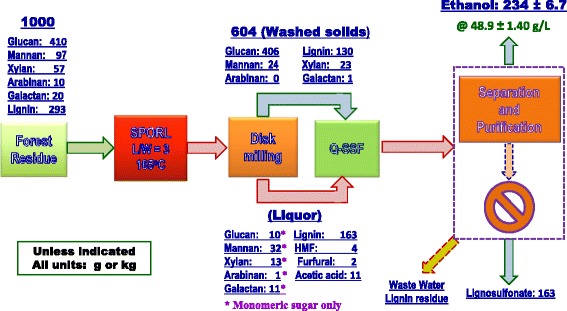
**An overall mass balance for high titer and yield ethanol production from a Douglas-fir forest residue pretreated by SPORL with “pH profiling” and acid injection delay time** ***t***_**i**_ **= 25 min.**

An overall mass balance for the pH profiling run with injection delay time *t*_i_ = 25 was conducted. Component yields from both the washed solids and pretreatment spent liquor were separately determined based on composition analyses of samples of washed solids and liquor (Figure 5). Actual liquor and solid separation was not conducted as shown in Figure 1, rather the whole slurries were used for simultaneous enzymatic saccharification and fermentation in ethanol production. Approximately 56% of the FS-10 lignin was solubilized to become lignosulfonate that can be recovered as a valuable co-product [17,25]. Substantial amounts of mannan (25%) and xylan (40%) were retained on the washed solids. Glucan dissolution was negligible. Percentage of major carbohydrates (glucan, mannan, and xylan) retained on solids was 80% compared with 74% for the control run. Final ethanol yield was 234 ± 6.7 kg (297 ± 9 L)/tonne FS-10 at 48.9 g/L, equivalent to 73.1 ± 2.1% theoretical ethanol yield based on the glucan, xylan, and mannan contents of FS-10. Comparing with 169 ± 32 kg (215 ± 42 L)/tonne FS-10 at 38.6 g/L, pH profiling increased ethanol yield by 38%.

## Conclusions

pH profiling in SPORL effectively reduced furan formation by as much as 70% depending on acid delay time when pretreating a Douglas-fir forest residue at a moderate temperature of 165°C for 75 min. pH profiling also reduced carbohydrate degradation. The improved carbohydrate yield in pretreated solids and reduced fermentation inhibitors with pH profiling enabled fermentation at 21% solids to result in a terminal ethanol titer of 48.9 ± 1.4 g/L and yield of 297 ± 9 L/tonne FS-10, which are substantially higher, i.e., by 27% in titer and by 38% in yield, than those of a control SPORL run without pH profiling. Based on the amount of furan formation, the estimated potential solid loading over 35% can be efficiently fermented without detoxification. SPORL was developed based on sulfite pulping which reduced risk for commercial scale-up. The demonstrated robust performance for high titer and high yield ethanol production with detoxification from one of the most recalcitrant feedstock—Douglas-fir forest residue—has commercial significance.

## Materials and methods

### Materials

Douglas-fir forest residue was collected from roadside piles resulting from a regeneration harvest in a Douglas-fir stand on Mosby Creek owned by Weyerhaeuser Company southeast of Cottage Grove in Lane County, OR. The residues were ground on January 8, 2013 by a Peterson horizontal drum-fixed hammer grinder (4710 Horizontal Grinder) using a combination of 76 and 102 mm grates. The harvested residues were shipped to Weyerhaeuser Company at Federal Way, WA. The moisture content was 43.9% measured at arrival. The collected residues were screened using a gyratory screen (Black-Clawson) equipped with a 1.75-inch diameter round hole punched plate top deck to remove oversized particles and a 1/8-inch clear opening woven wire bottom screen (6 wires/inch mesh) to remove fines. The screen reject fines were 7.6%. Physical fractionation through screening was proven effective to reduce ash and bark content to upgrade forest residue harvested using a chipping method [30]. The oversized particles were hammer milled at West Salem Equipment, which resulted near-zero oversized particles and 14.9% fines of the 9.8% initial screen oversize particles. The total rejection of fines was therefore at 9% with near zero-rejection of oversize particles. The accept residue or FS10 was then air-dried to moisture content of 15% and shipped to the USDA Forest Products Laboratory, Madison, WI. The chemical composition of FS10 is listed in Table 1.

The bark contents of the large fractions (>25.4 mm) were determined gravimetrically after manually separating the bark from the bark-free wood. Because of difficulties in separating bark from wood for the small fractions (<25.4 mm), the bark contents of the small fractions were calculated using the measured lignin and glucan contents of bark, bark-free wood, and the forest residue fractions as described in our previous study [30].

Commercial cellulase enzymes Cellic® CTec3 (abbreviated CTec3) were generously provided by Novozymes North America (Franklinton, North Carolina, USA). The cellulase activity was 217 filter paper unit (FPU)/mL as calibrated by a literature method [31]. Sodium acetate buffer, sulfuric acid, and sodium bisulfite were used as received from Sigma-Aldrich (St. Louis, MO). All chemicals were ACS reagent grade.

*S. cerevisiae* YRH400 is an engineered fungal strain for xylose fermentation [32]. The strain was grown at 30°C for 2 days on YPD agar plates containing 10 g/L yeast extract, 20 g/L peptone, 20 g/L glucose, and 20 g/L agar. A colony from the plate was transferred by loop to liquid YPD medium in a flask and cultured overnight at 30°C with agitation at 90 rpm on a shaking bed incubator (Thermo Fisher Scientific, Model 4450, Waltham, MA). The yeast seed concentration was monitored using optical density at 600 nm by a UV-vis spectrometer (Model 8453, UV-visible spectroscopy system, Agilent Technologies, Palo Alto, CA). The cultured medium was used to inoculate the fermentation culture.

### Pretreatment and pH profiling

SPORL pretreatments of Douglas-fir forest residue FS-10 with or without pH profiling were conducted in a 23 L rotating laboratory wood pulping digester using a dilute solution of sodium sulfite as described elsewhere [3,25]. In commercial practice, sulfite solution is prepared by bubbling SO_2_ into a hydroxide solution of desired pH [9]. Using pH profiling, the amount of SO_2_ applied initially should be based on the desired initial pH whether in the alkaline (pH 8–12), neutral (pH = 6–8), or bisulfite range (pH = 3–5). To simplify laboratory practice, aqueous sodium bisulfite solution of pH 4.0 was used together with sulfuric acid to adjust the pH of the sulfite solution. In the control SPORL run, sodium bisulfite together with sulfuric acid were all applied at the beginning (*t*_i_ = 0) to a desired initial solution pH of approximately 1.8 when measured at room temperature (Table 4). No additional acid was applied later in pretreatment. For the three pH profiling runs, the same amount of sodium bisulfite (*B* = 12 wt%) and sulfuric acid (*A* = 2.2 wt%; or 4 mL/L) on oven dry wood base as those of the control run were used, but acid was applied at varied delayed times of *t*_i_ during pretreatment (insert in Figure 1) through injection. The total pretreatment duration (*t*_T_ = 75 min), temperature (*T* = 165°C), and liquid volume to wood mass ratio (*L*/*W* = 3 L/kg) were also identical for all four runs with and without pH profiling (Table 4), so that fair comparisons can be made to demonstrate the advantages of the pH profiling concept. This set of pretreatment conditions was chosen based on previous SPORL optimization study using softwood [25] as well as a similar Douglas-fir forest residue [16].

**Table 4:** **List of pretreatment conditions of FS10 at 165°C using SPORL with and without pH profiling**

**Run label** ^**a**^	***t***_**T**_ **(min)**	**Initial pH**	**Sulfuric acid at** ***t*** **= 0 (wt.%)**	**Sodium bisulfite at** ***t*** **= 0 (wt.%)**	***t***_***i***_ **(min)**	**Sulfuric acid at** ***t***_***i***_ **(wt.%)**	**Final pH**	**L/W (L/kg)**
t0A4B12	75	1.79	2.2	12	0	0	1.45	3
t25A4B12	75	4.06	0	12	25	2.2	1.72	3
t35A4B12	75	4.06	0	12	35	2.2	1.40	3
t45A4B12	75	4.06	0	12	45	2.2	1.66	3

^a^txx stands for acid injection time in min, A4 stands for 4 mL of sulfuric acid in 1 L of initial pretreatment solution, and B12 stands for 12 wt.% of sodium bisulfite charge on wood.

At the end of the each pretreatment, an aliquot of spent liquor was taken for chemical composition analysis. The solids and spent liquor (neutralized) were together fed to a laboratory disk mill (Andritz Sprout-Bauer Atmospheric Refiner, Springfield, OH) for size reduction as described previously [33]. The disk plates had a pattern of D2B-505 and the disk plate gap was set at 1.0 mm. A sample of mill solids was washed for chemical composition analysis, yield determination, and enzymatic hydrolysis. The resultant FS-10 whole slurries, i.e., the complete mixture of disk-milled pretreated solids and spent liquor, were neutralized again to pH approximately 6.2 with solid lime for saccharification and fermentation.

### Enzymatic hydrolysis

Enzymatic hydrolyses of the washed solids were conducted at 2% (*w*/*v*) in 50 mL of 50 mM acetate buffer (pH 5.5) on a shake/incubator (Thermo Fisher Scientific, Model 4450, Waltham, MA) at 50°C and 200 rpm. An elevated pH of 5.5, higher than the commonly used pH 4.8–5.0, can significantly reduce nonproductive cellulase binding to lignin leading to enhanced lignocellulose saccharification [34-36]. The CTec3 loading was 15 FPU/g glucan. Aliquots of 1 mL enzymatic hydrolysate were taken periodically for glucose analysis after centrifugation at 13,000 *g* for 5 min. Each data point is the average of two analyses. The mean values and standard deviations (as error bars) from replicate runs were reported in plots.

### Quasi-simultaneous enzymatic saccharification and fermentation

Q-SSF of the pretreated FS-10 whole slurry was carried out in 250-mL Erlenmeyer flasks using a shaker/incubator (Thermo Fisher Scientific, Model 4450, Waltham, MA). Acetic acid/sodium acetate buffer (50 mM) of pH 6.0 was added into each of the pH-adjusted pretreated FS-10 whole slurry to conduct enzymatic saccharification using CTec3 at 24 FPU/g glucan at a total solid loadings of 21%. An elevated pH of 6.0, higher than the commonly used pH of 4.8–5.0, and lignosulfonate in the SPORL pretreatment spent liquor reduced nonproductive cellulase binding to lignin and enhanced lignocellulose saccharification [34-36]. The use of a higher CTec3 loading than that used for enzymatic hydrolysis was to facilitate solid liquefaction at high solids as Q-SSF was conducted on shaking bed without shear mixing. Liquefaction of the solids was conducted at 50°C and 200 rpm. The mixture was then cooled down to 35°C, and the shaker speed was reduced to 90 rpm and inoculated with 1 mL of yeast seed. The initial optical density at 600 nm of the yeast for all fermentation experiments was estimated at 5. No nutrients were applied during fermentation. Samples of the fermentation broth were taken periodically for analysis of monosaccharides, inhibitors, and ethanol. Reported results were the average of duplicate analyses. Replicate fermentation runs were conducted to ensure experimental repeatability. The standard deviations were used as error bars in plotting.

### Analytical methods

The chemical compositions of the untreated and pretreated lignocelluloses were analyzed as described previously [37]. All lignocellulosic samples were Wiley milled (Model No. 2, Arthur Thomas Co, Philadelphia, PA, USA) to 20 mesh (~1 mm) and hydrolyzed in two stages using sulfuric acid of 72% (*v*/*v*) at 30°C for 1 h and 3.6% (*v*/*v*) at 120°C for 1 h. Carbohydrates of the hydrolysates were analyzed by high-performance anion exchange chromatography with pulsed amperometric detection (ICS-5000, Dionex). Klason lignin was quantified gravimetrically [38]. For fast analysis, glucose in the enzymatic hydrolysates were measured using a commercial glucose analyzer (YSI 2700S, YSI Inc., Yellow Springs, OH, USA).

Monosaccharides (glucose, mannose, xylose, arabinose, and galactose) in the enzymatic hydrolysates and fermentation broths were determined using a HPLC system (Ultimate 3000, Thermo Scientific, Sunnyvale, CA) equipped with an RI (RI-101) and UV (VWD-3400RS) detector and BioRad Aminex HPX-87P column (300 × 7.8 mm) operated at 80°C. Double distilled water (d.d.w.) was used as eluent at a flow of 0.6 mL/min. Inhibitors (acetic acid, furfural and HMF) and ethanol were measured by the same HPLC system equipped with BioRad Aminex HPX-87H column (300 × 7.8 mm) operated at 60°C. Dilute sulfuric acid solution of 5 mM was used as eluent at a flow rate of 0.6 mL/min. All sample injection volumes were 20 μL. Samples were diluted in deionized water and filtered by a 0.22-μm filter prior to injection.

## Additional files

Additional file 1 Figure S1: Photos of different fractions of the as harvested Douglas-fir forest residue. **(a)** <3.2 mm. **(b)** 4.8–6.4 mm. **(c)** 9.5–12.7 mm. **(d)** 19.1–22.2 mm. **(e)** 28.6–31.8 mm. **(f)** The wet as-harvested Douglas-fir forest residue. **Figure S2:** Fractional (particle size) mass distributions of the as harvested Douglas-fir forest residue. **(a)** Oven dry and wet mass. **(b)** Bark mass.

## Abbreviations

SPORL: sulfite pretreatment to overcome the recalcitrance of lignocelluloses
CHF: combined hydrolysis factor that accounts for pretreatment temperature, time, and chemical concentrations
SED: substrate enzymatic digestibility defined as percent of solid substrate glucan enzymatically saccharified into glucose
EHGY: enzymatic hydrolysis glucose yield defined as enzymatic glucose yield as percent of glucan in the untreated lignocellulose
Q-SSF: quasi-simultaneous enzymatic saccharification and fermentation
FPU: filter paper unit
