# Supplementary material for: High titer and yield ethanol production from undetoxified whole slurry of Douglas-fir forest residue using pH profiling in SPORL
Source: Biotechnol Biofuels. 2015 Feb 15;8:22. doi: 10.1186/s13068-015-0205-3 (PMC4337254; doi:10.1186/s13068-015-0205-3)
Supplement: Additional file 1 Figure S1: — Photos of different fractions of the as harvested Douglas-fir forest residue. (a) <3.2 mm. (b) 4.8–6.4 mm. (c) 9.5–12.7 mm. (d) 19.1–22.2 mm. (e) 28.6–31.8 mm. (f) The wet as-harvested Douglas-fir forest residue. Figure S2: Fractional (particle size) mass distributions of the as harvested Douglas-fir forest residue. (a) Oven dry and wet mass. (b) Bark mass. [file 13068_2015_205_MOESM1_ESM.docx]

## Supporting Information

High Titer and Yield Ethanol Production from Undetoxified Whole Slurry of Douglas-fir Forest Residue using pH-Profiling in SPORL

**Jinlan Cheng^1,2^, Shao-Yuan Leu^3,2^, J.Y. Zhu^2*^, Rolland Gleisner^2^**

^1^ Jiangsu Provincial Key Lab of Pulp and Paper Science and Technology, Nanjing Forestry University, Nanjing, China

^2^ USDA Forest Service, Forest Products Lab, Madison, WI 53719, USA

^3^ Dept. Civil Environ. Eng., Hong Kong Polytechnic University, Kowloon, Hong Kong

* Corresponding author: J.Y. Zhu: [jzhu@fs.fed.us](mailto:jzhu@fs.fed.us), (608) 231 -9520

Fig. S1 Photos of different fractions of the as harvested Douglas-fir forest residue. (a) < 3.2 mm; (b) 4.8 – 6.4 mm; (c) 9.5 – 12.7 mm; (d) 19.1 – 22.2 mm; (e) 28.6 – 31.8 mm; (f) the wet as harvested Douglas-fir forest residue

| 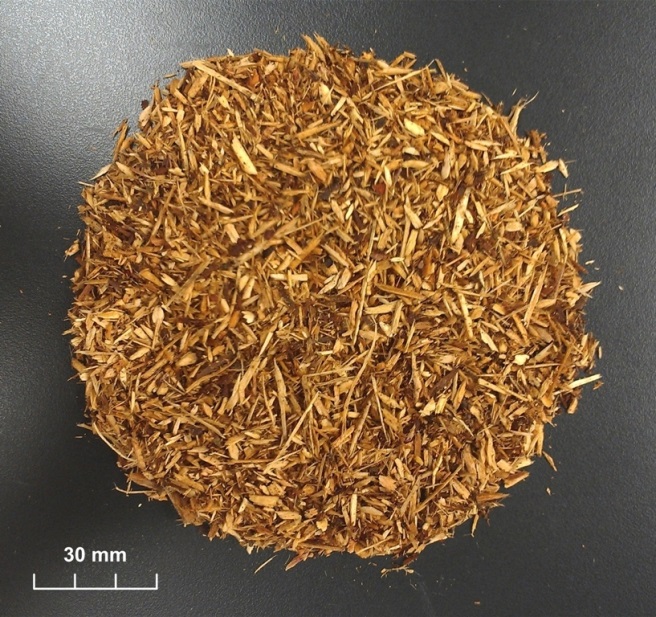  **(a)** | 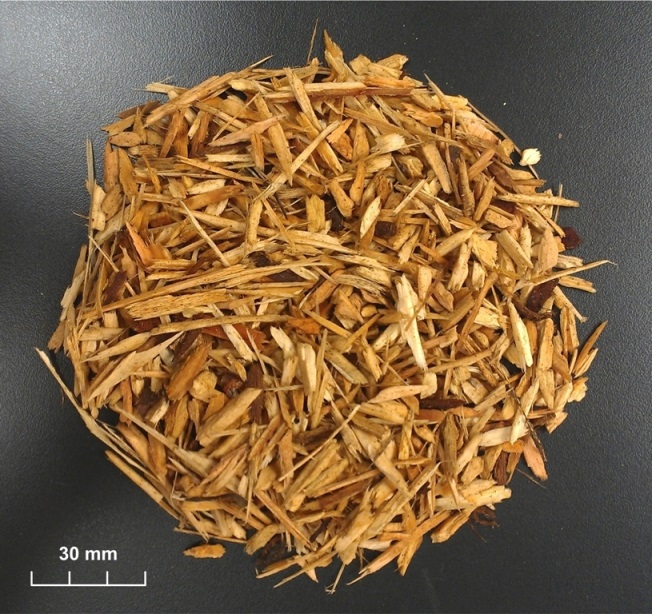  **(b)** | 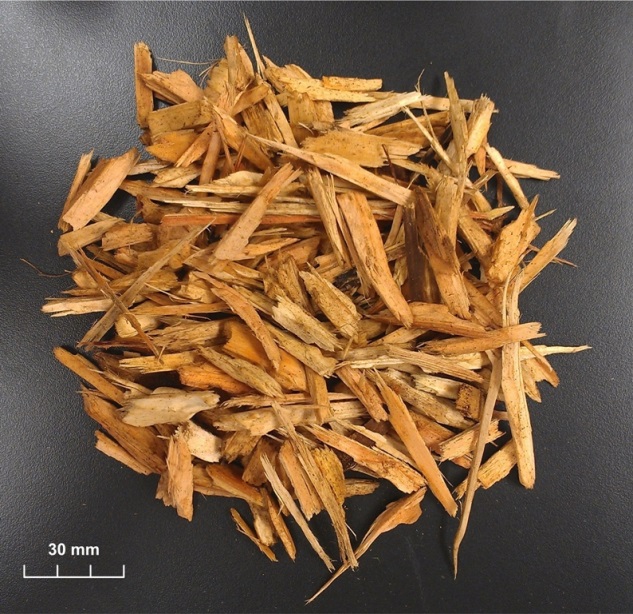  **(c)** |
| --- | --- | --- |
| 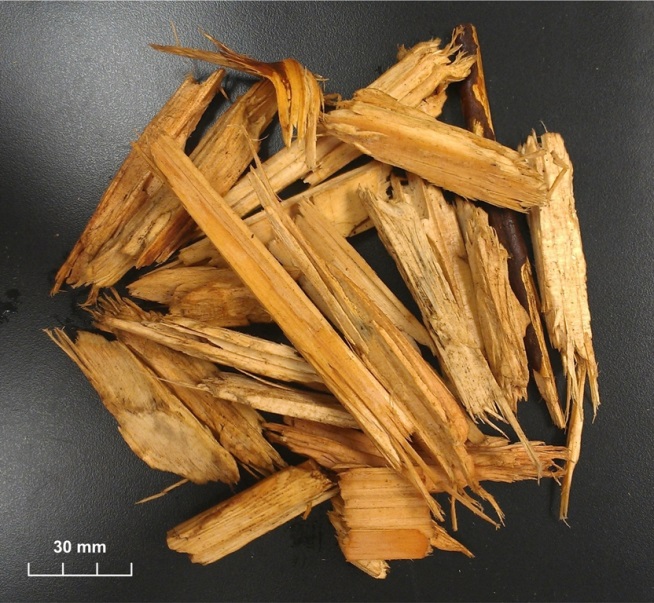  **(d)** | 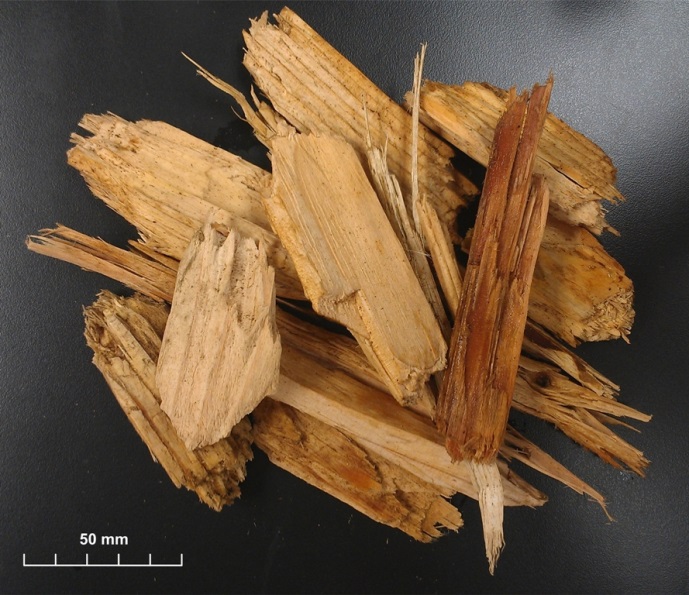  **(e)** | 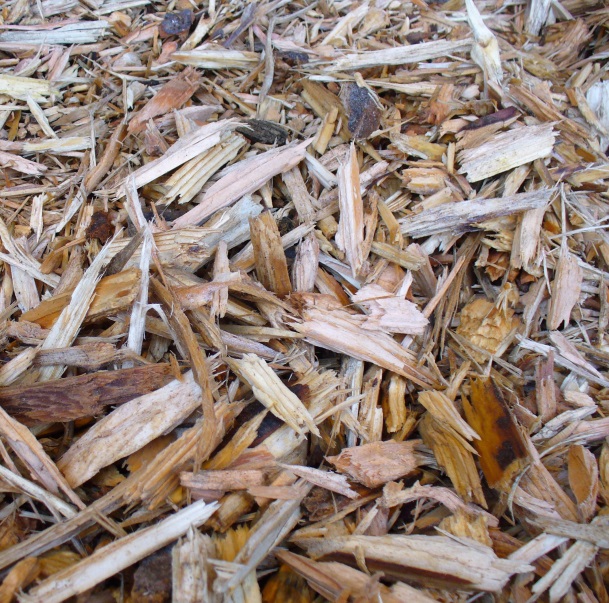  **(f)** |

Fig. S2 Fractional (particle size) mass distributions of the as harvested Douglas-fir forest residue. (a) oven dry and wet mass; (b) bark mass
